# Membranes with Surface-Enhanced Antifouling Properties for Water Purification

**DOI:** 10.3390/membranes7010013

**Published:** 2017-03-05

**Authors:** Nima Shahkaramipour, Thien N. Tran, Sankara Ramanan, Haiqing Lin

**Affiliations:** Department of Chemical and Biological Engineering, University at Buffalo, The State University of New York, Buffalo, NY 14260, USA; nimashah@buffalo.edu (N.S.); ttran7@buffalo.edu (T.N.T.); sramanan@buffalo.edu (S.R.)

**Keywords:** membrane fouling, surface modification, antifouling materials, state of water, poly(ethylene glycol), dopamine, zwitterions, fluoropolymers

## Abstract

Membrane technology has emerged as an attractive approach for water purification, while mitigation of fouling is key to lower membrane operating costs. This article reviews various materials with antifouling properties that can be coated or grafted onto the membrane surface to improve the antifouling properties of the membranes and thus, retain high water permeance. These materials can be separated into three categories, hydrophilic materials, such as poly(ethylene glycol), polydopamine and zwitterions, hydrophobic materials, such as fluoropolymers, and amphiphilic materials. The states of water in these materials and the mechanisms for the antifouling properties are discussed. The corresponding approaches to coat or graft these materials on the membrane surface are reviewed, and the materials with promising performance are highlighted.

## 1. Introduction

### 1.1. Membrane Technology

Wastewater reuse and seawater desalination are some of the key solutions in meeting the increasing demand for clean water. As an energy-efficient and low-cost technology, polymeric membranes permeate pure water and reject contaminants ranging from bacteria in microns to ions in angstroms [1,2,3,4,5]. For example, microfiltration (MF) membranes with pore sizes of 1–100 μm can remove microbes, cells and bacteria [1,3]; ultrafiltration (UF) membranes with pore sizes of 1–100 nm can remove small contaminants, such as proteins and viruses [3,6]; nanofiltration (NF) membranes having pore sizes of a few angstroms can remove divalent ions (e.g., Ca^2+^, Mg^2+^, Fe^2+^) and small molecules with a molecular weight of 200–1000 Da [3]; and reverse osmosis (RO) membranes with a dense selective layer that can desalinate brackish water and seawater [4,5,7]. The core of membrane technology is high performance membranes with high water permeance and high selectivity in a practical environment [1,2,3,4,5].

### 1.2. Membrane Fouling

**Figure 1:**
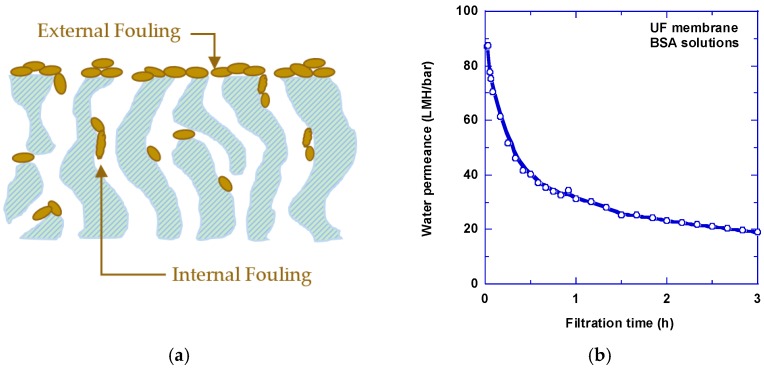
(**a**) Schematic showing external and internal fouling of membranes caused by contaminants from feed water; (**b**) an example of the decrease of water permeance over time caused by bovine serum albumin (BSA) in an ultrafiltration (UF) membrane (polyacrylonitrile (PAN)-50). The BSA concentration and pH of the solution were 0.3 g/L and 7.4, respectively [8].

Industrial membranes achieve high water permeance from an asymmetric structure comprising a thin skin layer exhibiting good separation properties on top of a thick support layer providing mechanical strength and low resistance to water transport. While the skin layer has been designed to be as thin as possible to increase water permeance, contaminants in the feed water may deposit and accumulate on the membrane surface (i.e., external fouling, as shown in Figure 1a), which would dramatically decrease water flux (as shown in Figure 1b) [1,3]. For MF and UF membranes, the contaminants may even block the internal pores (i.e., internal fouling, as shown in Figure 1a). The reduction of water permeance with time would not only cause inconvenience for practical operation, but also increase the operating costs because of the required membrane cleaning and replacement or increased energy input to compensate the permeance decrease [3]. Fouling is one of the key barriers preventing a more widespread adoption of energy-efficient membranes for industrial applications.

While MF and UF membranes suffer from both external and internal fouling due to their porous structure, NF and RO membranes experience mainly external fouling because of their tight skin layers [3]. Fouling can be reversible or irreversible [3]. Reversible fouling mostly derives from external fouling, and the weakly bound foulants can be removed by cleaning (e.g., backwashing) to recover water permeance [3,9]. On the other hand, when foulants have affinity towards the membrane and strongly attach to the membrane surface or pore wall, they cannot be removed by chemical or physical means, resulting in irreversible fouling [3]. Mitigation of fouling is an important step to retain water permeance and lower operating costs for membranes to be competitive with other technologies.

### 1.3. Surface Modification to Enhance Antifouling Properties

**Figure 2:**
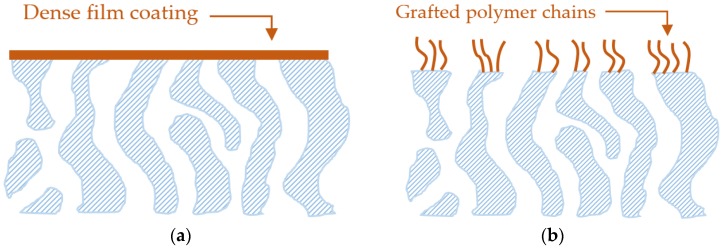
Schematic of membrane surface modification to enhance antifouling properties. (**a**) Coating of a thin nonporous dense film; and (**b**) grafting of polymer chains on top of membranes.

One effective strategy to mitigate membrane fouling is to enhance antifouling properties by surface modification. Two general approaches have been widely explored: coating a thin film on the membrane surface (as shown in Figure 2a) and grafting of polymer chains on the surface (as shown in Figure 2b) [1,3,10]. The materials for coating or grafting do not have affinity towards the foulants (e.g., proteins, emulsion and organic compounds), thus avoiding any favorable interactions between the foulants and membranes. The surface coating with a nonporous dense layer would also block the foulants from going through the skin layer and thus avoid internal fouling.

Besides the chemistry of the membrane surface, surface roughness and charges also affect the antifouling property [3]. For example, membranes with a rough surface are more susceptible to fouling because foulants can deposit in valleys on the membrane surface, preventing it from being removed by hydrodynamic force [1,11,12], though membranes with a nanopatterned surface have also been shown to improve antifouling properties [13,14]. Surface charge can promote fouling from foulants with counter-charges due to favorable electrostatic interactions. For example, the surface of NF and RO membranes (based on polyamides) is usually negatively charged, making it susceptible to fouling by positively-charged foulants, such as multivalent ions [1].

### 1.4. Outline of This Review

While improving antifouling properties, the coating or grafting would inevitably add another resistance layer for water transport and thus decrease water permeance. Therefore, there is great interest in designing coating or grafting materials as thin as possible with water permeance as high as possible to retain the high water permeance from the original membranes. This work reviews various materials with antifouling properties that have been coated or grafted onto the membrane surface to improve the antifouling properties of the membranes while retaining high water permeance. These materials can be separated into three broad categories, hydrophilic, hydrophobic and amphiphilic materials. The states of water in these materials are discussed since this is expected to influence water transport properties. The corresponding approaches to coat or graft these materials on the membranes are reviewed, and the materials with promising performance are highlighted in this short review.

## 2. Membrane Surface Modification Using Hydrophilic Materials

Hydrophilic materials create a hydration layer on the surface, which acts as a physical and energy barrier preventing foulants from attaching to the surface and thus reduces the fouling [1,15,16]. Therefore, membranes can be grafted or coated with hydrophilic materials to enhance hydrophilicity and thus antifouling properties. This section reviews several key hydrophilic materials explored for membrane surface modification, including poly(ethylene glycol) (PEG), polydopamine (PDA) and zwitterions. To elucidate the interactions between water and polymers, the states of water in polymers are first discussed.

### 2.1. States of Water in Polymers

There are three states of water sorbed by polymers, which can be determined using differential scanning calorimetry (DSC) [17]:

(1) Free water does not interact with polymers via hydrogen bonding or van der Waals interactions, and therefore, it exhibits the same melting temperature as bulk water.
(2) Freezable bound water forms due to weak interaction with polymers and/or capillary condensation in polymers, and therefore, its melting temperature is below 0 °C.
(3) Nonfreezing water strongly interacts with hydrophilic sites of polymeric chains via hydrogen bonding, and thus, it does not crystallize below 0 °C.

**Figure 3:**
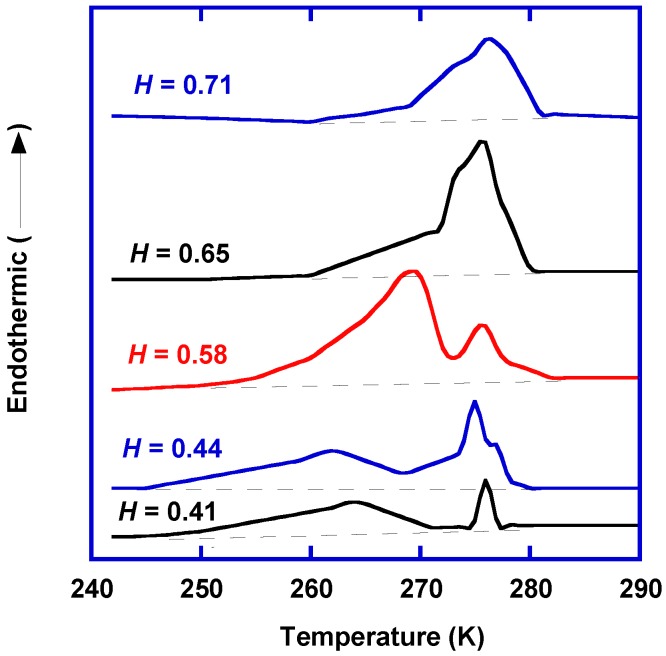
DSC heating curves for the swollen PVA with different degrees of water sorption (*H*). Adapted from [17].

Figure 3 depicts the DSC heating curves for water-swollen poly(vinyl alcohol) (PVA) at different degrees of water sorption, *H*, which is defined as [17]:

(1) $$H=\frac{m_{\mathrm{wet}}-m_{\mathrm{dry}}}{m_{\mathrm{wet}}}$$

where *m*_wet_ and *m*_dry_ are the mass of swollen polymer and dry polymer, respectively. For the swollen PVA with *H* ≤ 0.58, two distinct endothermic peaks at 273 K and <273 K were observed, which indicated the presence of free water and freezable bound water, respectively. For the swollen PVA with *H* > 0.58, there was only one broad peak for freezable water, including free and freezable bound water. The nonfreezing water did not crystallize, and thus, it cannot be detected using DSC.

The amount of freezable water (*w*_fw_) including free water and freezable bound water can be calculated from the peak area from the DSC curves using the following equation:

(2) $$w_{fw}=\Delta H_{fw}/\Delta H_{W}$$

where *ΔH*_fw_ is the peak area (J/g) and *ΔH*_W_ is the melting enthalpy of pure water at 273 K (334 J/g) [18]. The amount of nonfreezing water can be calculated by combining Equations (1) and (2).

**Figure 4:**
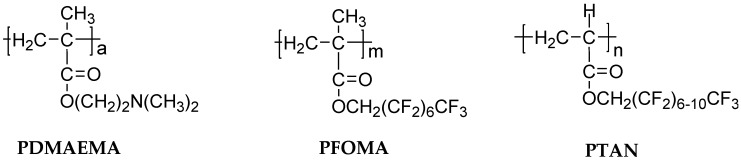
Chemical structure of hydrophilic poly(2-dimethylaminoethyl methacrylate) (PDMAEMA) and hydrophobic poly(1,1′-dihydroperfluorooctyl methacrylate) (PFOMA) and poly(1,1,2,2-tetrahyfoperfluorooctyl acrylate) (PTAN) [19].

The states of water in polymers can significantly affect the transport properties of water and salts. As an example, block copolymers containing hydrophilic poly(2-dimethylaminoethyl methacrylate) (PDMAEMA), and hydrophobic poly(1,1′-dihydroperfluorooctyl methacrylate) (PFOMA) and poly(1,1,2,2-tetrahyfoperfluorooctyl acrylate) (PTAN) (as shown in Figure 4) were prepared, and the effect of copolymer composition on water sorption and NaCl diffusion coefficient was studied [19]. Increasing the content of hydrophilic PDMAEMA in copolymers increased the total water sorption [19,20].

**Figure 5:**
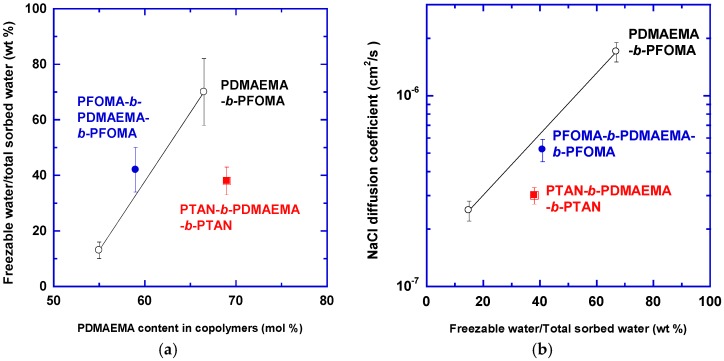
(**a**) Effect of PDMAEMA content on percentage of freezing water and (**b**) the effect of the percent freezing water on the NaCl diffusion coefficient in block copolymers at 25 °C. Adapted from [19].

Figure 5a shows that the percentage of freezable water in the total absorbed water increased with increasing the PDMAEMA content in the copolymers [19]. Figure 5b shows that there was a direct relationship between the NaCl diffusion coefficient and the percentage of freezable water in the total absorbed water. Increasing the amount of freezable water increased NaCl diffusivity, presumably because salts can only be dissolved in the freezable water, which formed channels for salt diffusion. The nonfreezable water interacted strongly with the hydrophilic sites and may not be accessible for NaCl sorption or diffusion.

We speculate that the relative amount of water at different states (instead of the total absorbed water) may play a role in forming the hydration layer and influencing antifouling properties. For example, the nonfreezable water may exert a greater energy barrier for foulants to attach to the surface than the free water. However, there is a lack of systematic study elucidating any relationship between the amount of water at different states and antifouling properties.

### 2.2. Poly(ethylene glycol)-Based Coatings

Poly(ethylene glycol) (PEG) has demonstrated good antifouling properties towards proteins and oil emulsions [15]. These contaminants such as proteins can be considered as an infinite length block placed parallel to the surface and perpendicular to the PEG chains [15,21,22]. The PEG chains are then compressed and exhibit repulsive elastic force to resist the protein adhesion [10,21,22]. More importantly, PEG can form hydration layers due to the hydrogen bonds with water, which act as energy barriers for protein adhesion [15,22,23]. Therefore, PEG-based materials have been widely used to modify the membrane surface to enhance antifouling properties in the following two approaches.

The first approach is to design PEG-based polymers that are insoluble in water, which can then be directly coated on top of membranes, as shown in Figure 2a [24,25,26,27,28]. For example, PEBAX^®^ block copolymers containing PEG and water-insoluble polyamides were coated on top of MF, UF and RO membranes [24,29]. These copolymers are microphase-separated and have a bicontinuous structure, and thus, water can permeate through the PEG phase in the thin films. Crosslinked PEG prepared from poly(ethylene glycol) diacrylate (PEGDA) was also applied to coat on UF membranes [26,30]. These thin film coatings increased membrane antifouling properties, and the modified membranes exhibited much higher water flux than the pristine membrane when tested under fouling conditions, though the coated layer increased the water transport resistance. Longer PEGDA chain length and higher PEGDA content in coating solutions resulted in lower BSA adhesion [27].

The second approach is to graft PEG chains on the membrane surface, as shown in Figure 2b [1]. In this approach, PEG-based materials contain functional groups that can be covalently bound to the membrane surface [1]. For example, poly(ethylene glycol) dimethacrylate was grafted onto the membrane surface via surface-initiated atom transfer radical polymerization (ATRP) [31]. Amine functionalized PEG (PEG-NH_2_) was grafted on the membrane surface after the deposition of polydopamine (PDA), a bio-adhesive, which will be discussed later in this work [6,32]. The modified membranes were less susceptible to fouling by BSA than the unmodified ones [6,32]. Increasing PEG-NH_2_ grafting density (i.e., the number of PEG chains per area) by either increasing grafting time or PEG concentration in grafting solutions reduced the adhesion of BSA [6].

### 2.3. Polydopamine

Mussels can easily attach to any surface, including hydrophilic and hydrophobic ones. The key composition of adhesion proteins for mussels is dopamine [33,34], which has been demonstrated to be an effective bio-inspired building block for surface coating. For example, dopamine can be coated by simple dipping in an aqueous solution on a variety of polymeric substrates, such as hydrophobic polysulfone (PSf), polytetrafluoroethylene (PTFE) and polydimethylsiloxane (PDMS) [34]. Coated layer thickness increased with increasing immersion time and can be up to 50 nm after a 24-h immersion [6,34]. The dopamine coating significantly increased the hydrophilicity of substrates, and therefore, dopamine has emerged as an attractive platform to modify the membrane surface to enhance antifouling properties [1,35].

#### 2.3.1. PDA Structure

**Figure 6:**
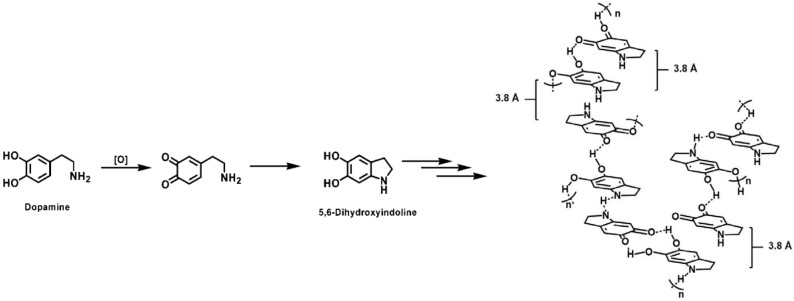
A possible mechanism for the oxidation and polymerization of dopamine to form insoluble aggregates [1,36,37]. Reproduced with permission, ACS Publications, 2012.

While dopamine is believed to be oxidized by oxygen in the air and then forms PDA, which easily adheres to substrates, the detailed mechanism and the structure of PDA are still under debate. Freeman and colleagues propose that dopamine is first oxidized by oxygen to form dopaquinone and then 5,6-dihydroxyindoline (DHI), as shown in Figure 6 [36]. This proposed model is based on the Raper–Mason model developed to explain the oxidation and polymerization of tyrosine (which has similar structure to dopamine) to form melanins (i.e., polyphenolic molecules) [36].

There are two main theories for the self-polymerization of DHI to PDA, covalent linkages and non-covalent linkages [1,36,37]. In the first model, DHI forms new covalent bonds between heterocycles of varying oxidation state due to nucleophilic-electrophilic interactions [1,36,38,39]. In the second model (as shown in Figure 6), PDA is formed through the non-covalent bonds, such as charge transfer, π-stacking, and hydrogen bonding between monomers, which are evidenced by solid state nuclear magnetic resonance (NMR) and electron paramagnetic resonance (EPR) spectroscopy, powder X-ray diffraction and Fourier transfer infrared (FTIR) spectroscopy [36,37]. Hydrogen atoms linked to the carbocyclic core confirm the non-covalent linkages between monomers, which was also observed for other materials with similar molecular architectures, such as quinhydrones, supramolecular polymers, proteins, etc. [36,37]. Both models predict that a robust and stable PDA aggregates on substrates, while the non-covalent bond model is more consistent with the chemistry for similar molecular architectures [1].

#### 2.3.2. PDA Coating on Membrane Surface for Water Purification

**Figure 7:**
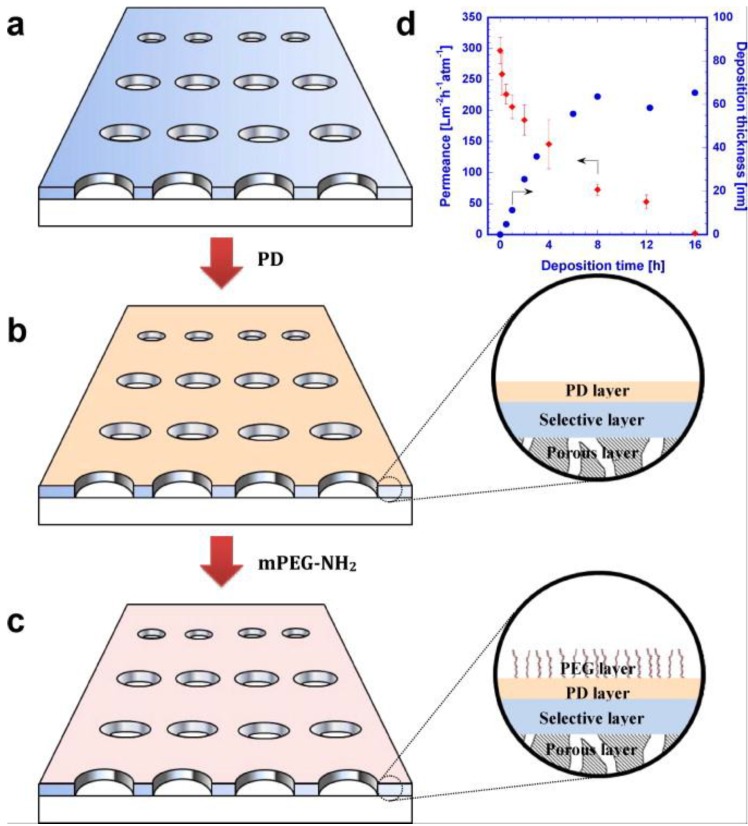
Schematic of polydopamine (PDA)-*g*-PEG coating on microporous membranes. (**a**) A polysulfone UF membrane; (**b**) a PDA-coated membrane; (**c**) a PDA-*g*-PEG modified membrane; and (**d**) the effect of PDA deposition time on the layer thickness and water permeance in polysulfone (PSf) membranes [32]. Reproduced with permission, Elsevier B.V., 2012.

With good hydrophilicity, great adhesion to a variety of polymers and facile coating using aqueous solutions, dopamine has been widely explored to enhance the surface hydrophilicity of membranes, aiming to reduce fouling [6,32,40,41,42,43,44]. For example, PDA has been successfully coated on MF, UF and RO membranes when special procedures were developed to ensure the exposure of the membrane surface to oxygen during the coating [6,32,45]. The PDA coating decreased the water contact angle or increased the surface hydrophilicity for all membranes, and the thickness of the PDA layer increased with the coating time, as shown in Figure 7. Water permeance decreased with increasing coating time, which is ascribed to the added transport resistance and decreased pore size and porosity due to the PDA coating [6]. The PDA coating was demonstrated to decrease the adhesion of BSA and oil emulsions. Moreover, due to the simplicity in operation, the PDA coating was scaled up for industrial spiral-wound modules containing UF membranes and evaluated for wastewater treatment [32,41,42]. Besides flat sheet membranes, the PDA coating was also successfully applied to hollow fiber membranes by solution dipping [46].

The effect of PDA coating on the pore size in UF membranes has been examined. For example, when a PSf UF membrane, with molecular weight cutoff (MWCO) of 20 kDa, was coated by dopamine for 75 min, the pore size decreased to that of unmodified PS-10 with MWCO of 10 kDa [47]. When a polyethersulfone (PES) membrane (PES-20), with MWCO of 20 kDa, was coated for 5 min and 30 min, the pore size became equivalent to that of PES-10 and PES-5, respectively. When the membranes with a similar pore size were tested with wastewater containing oil/water emulsion in a constant permeate flux crossflow system, the PDA-coated membranes demonstrated lower transmembrane pressure than the unmodified ones, indicating the enhanced antifouling properties by PDA coating [47,48].

The pH value of the dopamine solution is critical to obtain good PDA coating [40]. For example, RO membranes showed essentially the same water permeance when coated using a dopamine solution with a pH of 5 and much lower permeance when coated using solutions with a pH of 8 and 11 due to the thicker PDA layer [40]. This behavior is ascribed to the requirement of an alkaline condition for dopamine to polymerize [49]. Nevertheless, the modified membranes under all pH values exhibited enhanced antifouling properties, as the long-term water flux was higher than the uncoated analogs in an oil/water crossflow filtration test [40].

#### 2.3.3. PDA as a Bio-Glue to Coat the Second Layer on Membranes

While strongly adhering to substrates, PDA behaves as a versatile immobilization platform to covalently anchor a second layer, which can be a self-assembled monolayer or grafted polymer chains with superior hydrophilicity [34]. The catechols in PDA can react with thiol or amine groups through Michael addition and Schiff base reactions [34,50]. For example, thiol-terminated methoxy-poly(ethylene glycol) (mPEG-SH) can be coated on top of PDA, and the mPEG-SH coating decreases the cell adhesion, demonstrating the enhanced antifouling properties [34]. The PDA layer can also be used to anchor zwitterionic materials to enhance surface hydrophilicity, which will be discussed in Section 2.4.

As shown in Figure 7, the PDA-coated surfaces can be grafted with PEG-NH_2_ to further improve antifouling properties in membranes [32]. The grafted PEG layer decreased the BSA adhesion and water permeance due to the additional mass transfer layer. On the other hand, when tested with an oil/water emulsion using a constant pressure crossflow system, the long-term water flux in the PDA-*g*-PEG-modified MF and UF membranes was higher than that in the uncoated ones. For NF and RO membranes, the flux of PDA-*g*-PEG-modified membranes remained constant [32]. The membranes were also applied to short-term batch tests of protein and bacteria adhesion, and the modified membranes showed less adhesion of proteins and bacteria than the unmodified ones [41]. However, in the long-term biofouling testing, the modified membranes did not exhibit any improvement in antifouling properties [41]. This trend had also been shown for NF and RO membranes [51], presumably because the surface modification cannot fully prevent biofilm formation in the long run. Thus, periodical membrane cleaning was still needed. The PDA-*g*-PEG coating may reduce the frequency of the membrane cleaning and, thus, lower operating costs.

The surface modification using PDA-*g*-PEG was also scaled up for commercial spiral-wound modules containing UF and RO membranes, which were installed in a pilot skid to treat produed water from hydraulic fracturing operation [42]. The PDA-*g*-PEG-modified UF membrane modules showed improved flux and decreased transmembrane pressure compared with the unmodified ones. On the other hand, the coated RO modules did not show higher water permeance than the unmodified ones, presumably due to the cleaner feed stream for the RO membrane system than the UF membrane system. However, the modified RO modules demonstrated higher salt rejection than the unmodified ones, presumably due to the caulking of minor defects in the RO membranes [42].

PDA can also be used to graft amine-containing materials such as poly(ethyleneimine) (PEI) on top of the PDA layer. For example, NF membranes with a positive surface charge were modified using PDA-*g*-PEI [52]. The modified membranes exhibited a stable water flux and rejection rate of methylene blue over a long-term test for wastewater treatment [52].

As another example, the PDA coating can be exploited to anchor nanoparticles on the membrane surface [53]. For example, TiO_2_ nanoparticles were anchored to PDA through the interactions with free hydroxyl groups in PDA. Introducing the hydrophilic TiO_2_ nanoparticles on the membrane surface increased surface hydrophilicity without decreasing pure water flux [53].

#### 2.3.4. Dopamine-Like Materials

**Figure 8:**
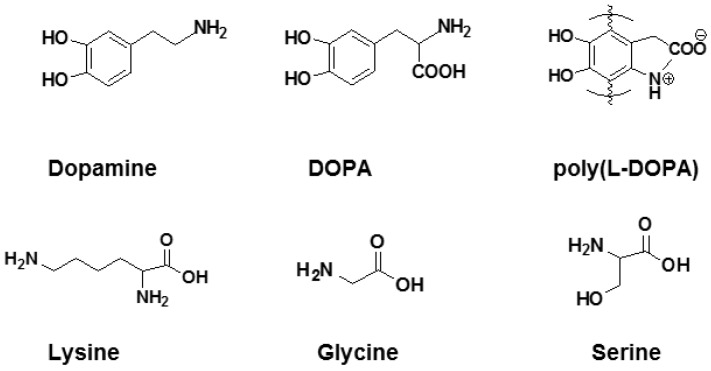
Chemical structure of dopamine, 3,4-dihydroxyphenylalanine (DOPA) [1], poly(3-(3,4-dihydroxyphenyl)-l-alanine) (poly(l-DOPA)) [56], lysine, glycine and serine [57].

There exist other catecholamines with structures similar to PDA, such as 3,4-dihydroxyphenylalanine (DOPA), which also demonstrate good stability and durability of coatings [36]. As shown in Figure 8, DOPA contains a pendant carboxylic acid moiety to enhance adhesion on substrates [54]. Surface modifications using poly(DOPA) with more hydrogen bonding units than PDA can result in a more stable layer with greater resistance to chemical degradation than PDA [37]. For example, both PEG and DOPA were used to coat TiO_2_ substrates, which significantly decreased the adsorption of serum proteins, indicating the excellent enhancement of antifouling behaviors [55].

As a PDA-like compound, 3-(3,4-dihydroxyphenyl)-l-alanine (l-DOPA, as shown in Figure 8) was utilized to modify the surface of a RO membrane [56]. Since l-DOPA comprises both carboxyl (with a negative charge) and amine (with a positive charge) functional groups, it is regarded as a zwitterionic material [56]. Water contact angles decreased from ~55° to ~28° after 4 h of l-DOPA coating. The modified RO membranes exhibited much lower BSA adsorption than the unmodified ones. Interestingly, the l-DOPA coating also enhanced water permeance, due to the improved surface hydrophilicity, and retained the high salt rejection rate.

Natural amino acids with structures similar to dopamine have also been explored. Figure 8 shows three representative amino acids, such as lysine, glycine and serine. Polyacrylonitrile (PAN) UF membranes functionalized with carboxylic acid groups were grafted with these amino acids on the surface [57]. The amino groups reacted with carboxylic acid groups and thus attached to the membrane surface, which was confirmed by the X-ray photoelectron spectroscopy (XPS) study. Though the amino acid modification did not decrease water contact angles, the lysine-grafted membranes showed decreased adsorption of BSA and lysozyme in the static exposure experiments. In a crossflow system test with BSA solutions, the lysine-grafted membranes showed stable water flux, indicating negligible fouling.

Despite the extensive work on the PDA coating for membrane applications, there are few studies exploring dopamine-like structures with better hydrophilicity, stronger adhesion capability to substrates and more versatile reactions to anchor the second layer than the PDA, which would be useful to expand the platform of bio-glues for membrane applications.

### 2.4. Zwitterionic Materials

**Figure 9:**
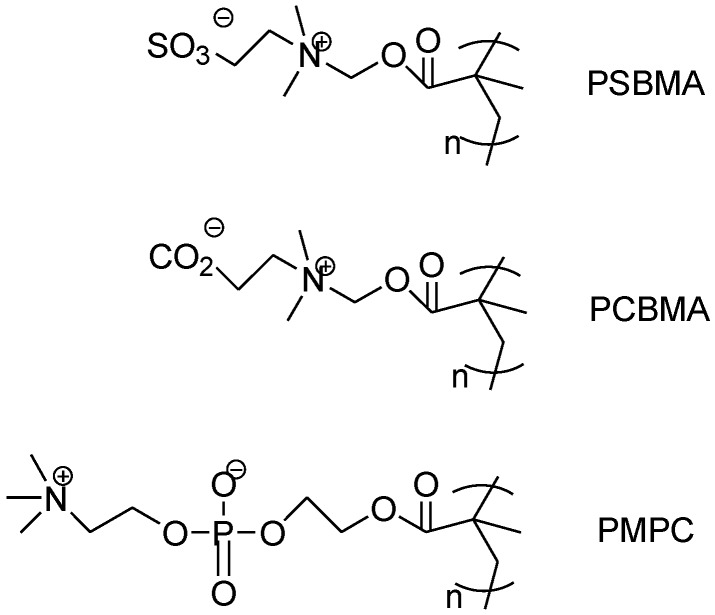
Chemical structure of three representative polybetaines, poly(2-methacryloyloxyethyl phosphorylcholine) (PMPC), poly(carboxybetaine methacrylate) (PCBMA) and poly(sulfobetaine methacrylate) (PSBMA) [58].

Zwitterionic materials contain both positively- and negatively-charged groups and have neutral charge [58,59,60,61]. Figure 9 shows typical zwitterionic polymers containing betaine groups, which have been widely investigated as antifouling materials to decrease protein adhesion. These polybetaines comprise a cation of quaternary ammonium and an anion of phosphate, sulfonate or carboxylate. The charged groups interact with water through electrostatic forces to form tight hydration layers or physical and energy barriers preventing proteins from attaching to the polymers. On the other hand, the positively- and negatively-charged groups are closely connected together, and the polymer chains show a neutral charge when exposed to proteins in the size of nanometers, avoiding any favorable interactions with proteins of any specific surface charges.

#### 2.4.1. Superhydrophilicity in Zwitterionic Materials

**Figure 10:**
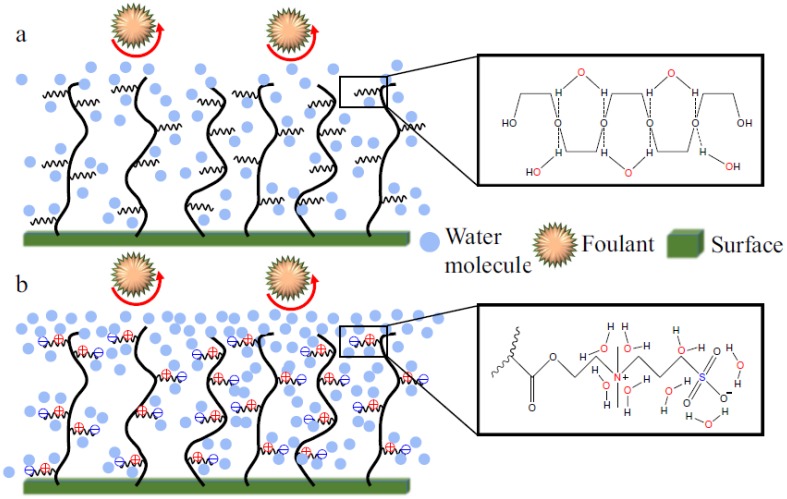
Schematics comparing the interactions of zwitterions and PEG with water. (**a**) One PEG repeat unit forming a hydrogen bond with one water molecule; (**b**) one zwitterion chain interacting with eight water molecules through electrostatic forces [58]. Reproduced with permission, Elsevier B.V., 2016.

Zwitterions have emerged as a new generation of materials with superior antifouling properties. Figure 10 compares the mechanism of antifouling in zwitterions with that in PEG-based materials [58]. PEG comprises one oxygen atom in each repeat unit (–CH_2_CH_2_O–) that can form a hydrogen bond with only one water molecule, while zwitterions can form electrostatic forces with up to eight water molecules, which also being stronger than the hydrogen bonding in PEG/water systems. Therefore, zwitterions can be more hydrophilic than PEG [58,62]. A study of copolymer hydrogels prepared from PEGDA and zwitterionic monomer, such as sulfobetaine methacrylate (SBMA), exhibit greater hydrophilicity than those from PEGDA [61,63,64]. Similar to PEG, zwitterionic chains have elastic forces against foulants when they compress the chains, which also contributes to antifouling properties [65].

Zwitterions also demonstrate more stable antifouling properties than PEG-based materials in high salinity environments. PEG is amphiphilic, and the high salinity can lead to the collapse of PEG chains, decreasing the surface hydrophilicity. However, zwitterionic chains tend to maintain an open structure and strong hydration layers in the presence of salts [66,67].

#### 2.4.2. Surface Coating Using Dense Zwitterions

Zwitterionic materials can be directly coated onto the membrane surface to enhance hydrophilicity, as shown in Figure 2a [58]. For example, copolymers of PTFE-*co*-SBMA were synthesized and coated on top of UF membranes [68]. The copolymers can self-assemble and form nano-channels with diameters of about 1 nm. The modified UF membranes exhibited higher water flux than the unmodified ones. When the membranes were tested with water containing 1 g/L BSA and 1500 mg/L oil, the modified one showed only 4% decline in water flux, demonstrating good antifouling properties derived from the zwitterionic coating [68].

Thin films of zwitterionic polymers can also be formed on the membrane surface by in situ polymerization, such as initiated chemical vapor deposition (iCVD) [69,70,71]. In this way, an initiator and monomer in the vapor phase flow into a chamber at high temperatures. The initiator decomposes and attaches to the membrane surface kept at low temperatures, which initiates polymerization to form thin films. This technique allows the use of substrates with nonplanar geometries and also a vast variety of substrates, which cannot be coated in liquid phase [70]. For example, poly[*N*,*N*-dimethyl-*N*-methacryloxyethyl-*N*-(3-sulfopropyl)-*co*-2-(dimethylamino)ethyl methacrylate-co-ethylene glycol dimethacrylate] (PDDE) thin films were synthesized via iCVD followed by a reaction with 1,3-propane sultone in vapor phase to obtain zwitterionic coating [70]. When the modified surfaces were tested with BSA, humic acid and sodium alginate, they showed less foulant adhesion than the bare ones, indicating an improvement in antifouling properties derived from the zwitterions.

#### 2.4.3. Surface Grafting of Zwitterions

Zwitterions can also be grafted from the membrane surface, i.e., zwitterionic monomers are polymerized from the surface functionalized with initiators, as shown in Figure 2 [58,72]. Depending on the type of initiators, different polymerization can be utilized, such as photo-initiated, ozone-initiated, plasma-initiated and physisorption radical graft polymerization [58]. For example, poly(2-methacryloyloxyethyl phosphorylcholine) (PMPC) was grafted from a PES membrane via photo-initiated polymerization, and the modified surface exhibited much less accumulation of bacteria than the unmodified one [73]. Moreover, [3-(methacryloylamino)propyl]-dimethyl(3-sulfopropyl) ammonium hydroxide (MPDSAH) was grafted from the polypropylene membrane through UV-irradiated polymerization, and the modified membrane showed less BSA adhesion than the unmodified surface. A high flux recovery ratio of 90% was achieved for the modified membranes when treating BSA solutions [74].

To better control the grafting density, zwitterionic monomers can be polymerized using living polymerization, such as ATRP and reversible addition-fragmentation chain-transfer polymerization (RAFT) [58]. For example, PSBMA and poly(carboxybetaine methacrylate) (PCBMA) were grafted from glass using an initiator of 2-bromo-2-methyl-*N*-3-[(trimethoxysilyl)propyl]propanamide (BrTMOS) and the ATRP method, and the modified glass demonstrated less adsorption of protein and mammalian cells than the unmodified one [75]; 3-dimethyl (methacryloyloxyethyl) ammonium propane sulfonate (DMAPS) was grafted from cellulose membranes using RAFT polymerization, and the modification decreased the adhesion of *Escherichia coli* and HeLa cell [76].

**Figure 11:**
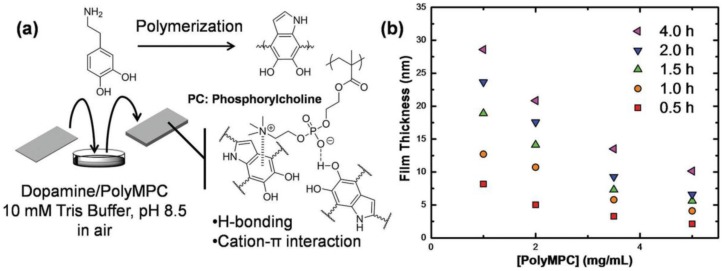
(**a**) Schematics showing the PDA-*g*-PMPC coating and the mechanism of the PDA-PMPC interactions; and (**b**) the effect of PMPC content and coating time on the coating thickness. Reproduced with permission, John Wiley & Sons, Inc., Hoboken, NJ, USA, 2016. [78].

Zwitterions can also be grafted to the membrane surface via a glue, such as PDA [77,78]. Figure 11 shows an example of PDA-*g*-PMPC coating on a variety of substrates [78]. PMPC interacted with PDA through non-covalent linkages, including phenol-phospholipid hydrogen bonding and cation-π interactions [78,79]. The coating increased surface hydrophilicity (as indicated by the decreased water contact angle) and significantly decreased the *E. coli* adhesion [78].

Computational modeling has been performed to elucidate the determining factors in achieving superior antifouling properties derived from zwitterions, such as PSBMA-grafted surfaces. The antifouling properties can be affected by packing density, grafting coverage, polymer chain conformation and chemistry [80]. For example, protein fouling resistance increased with increasing polymer chain length and grafting coverage [59,81].

Due to the versatility of ionic groups in the zwitterionic materials, they present a promising platform to improve antifouling properties for membranes. Currently, most studies are focused on three common materials, such as PSBMA, PMPC and PCBA. More thorough studies in the structure and property relationship are needed to design the most suitable zwitterions for different applications [82,83], since real wastewaters may contain various foulants, such as algae, bacteria and proteins [58].

## 3. Membrane Surface Modification Using Hydrophobic or Amphiphilic Materials

### 3.1. Fluoropolymers

Low energy surfaces have been demonstrated to be resistant to the adhesion of bacteria, micro- and macro-molecules and precipitated salts, such as CaSO_4_ [10,84,85]. Additionally, due to their non-sticking nature (i.e., weak binding forces), the accumulated matters on the low energy surface can be easily washed off. Therefore, there is great interest in designing a thin coating layer with low surface energy of 10–20 mN/m [10,22]. Two most common materials that exhibit the desired surface energy are fluorine-based and silicone-based materials. Most work is focused on the fluorinated materials, as discussed below.

Fluoropolymers have exposed CF_3_ moieties on the surface, resulting in the low energy and low adhesion (non-sticking) properties [22]. For example, poly(perfluoroacrylate) was coated on glass substrate, and the coating exhibited a low surface energy (<13 mN/m) and relatively smooth surface (i.e., roughness < 6 nm) [86,87]. The coating improved resistance towards the adhesion of bacteria. Increasing the length of the fluorinated side chains decreased the surface energy and improved resistance to the bacteria adhesion. In another study, copolymers prepared from *n*-alkyl methacrylate with different lengths and 2-perfluorooctylethyl methacrylate decreased the adsorption of proteins, such as fibrinogen, linearly with increasing CF_3_/CF_2_ ratio [88]. At a CF_3_/CF_2_ ratio of 0.26, there was no fibrinogen adhesion on the surface observed. Such resistance was attributed to the closely-packed array of CF_3_ groups, which reduced the surface energy. 

Perfluoropolyethers (PFPEs) are another class of perfluoropolymers that has been widely studied. For example, random PFPE-graft terpolymers reduced the settlement while enhancing the removal of macroalga *Ulva* spores [89]; crosslinked PFPEs were also prepared from dimethacrylate and showed low surface energy (~14 mN/m) and low settlement of zoospore [90].

Membranes can also be directly fluorinated to enhance antifouling properties [91]. The surface fluorination of polyamide-based NF membranes reduced the surface energy from 60.0 to 44.4 mN/m. When tested with BSA solutions, the fluorinated membranes showed much lower flux reduction (8.0%) and higher flux recovery (98.5%) than the unmodified ones [91].

### 3.2. Amphiphilic Polymers

While both hydrophilic coatings (based on PEG, PD, and zwitterions) and non-sticky coatings with low surface energy (such as fluorinated polymers) suppress the adsorption of proteins and organisms, amphiphilic materials comprising both hydrophilic and non-sticky components have been explored to further enhance antifouling properties [92]. For example, crosslinked networks of hyperbranched fluoropolymers and PEG at various compositions were prepared [92]. When PEG content increased from 14 wt %–55 wt %, the water contact angle reduced from 101° to 74° and the surface-free energy increased from 22 mN/m–35 mN/m, since PEG has a higher surface energy >40 mN/m and a lower water contact angle than fluoropolymers [92]. The surface treated with amphiphilic polymers showed resistance towards the adsorption of proteins such as BSA. Furthermore, the settlement of *Ulva* spores was lower on a coated glass than on an uncoated one [92].

Thin films of amphiphilic materials can be coated on membrane surfaces via chemical vapor deposition (CVD) [93]. For example, when copolymers of hydrophilic hydroxyethyl methacrylate (HEMA) and hydrophobic perfluorodecyl acrylate (PFA) were deposited on a RO membrane, the adhesion of *E. coli* bacteria on the RO membrane surface was reduced [93]. More importantly, the surface modified by the copolymers showed less BSA adhesion than that modified by either HEMA or PFA, suggesting a synergistic effect of HEMA and PFA in amphiphilic copolymers [94].

**Figure 12:**
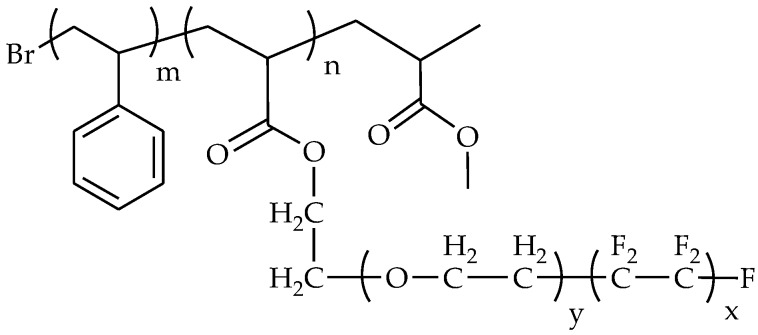
Chemical structure of poly(ethoxylated fluoroalkyl acrylate)-*b*-polystyrene comb-like block copolymer with amphiphilic side chains [95].

Figure 12 shows the chemical structure of block copolymers of polystyrene and polyacrylate with amphiphilic side chains consisting of both PEG and perfluoroalkyl groups [95]. This comb-like block copolymer was spin-coated on a silicon wafer and tested against alga *Ulva* and cells of a diatom *Navicula* [86,95]. The surface modification decreased settlement and increased the removal of *Ulva* and *Navicula*, compared with the uncoated one. Though the settlement of diatom on the amphiphilic surface was comparable to polydimethylsiloxane (PDMS), the diatom removal rate from the amphiphilic surface was about eight-times higher than PDMS, which is ascribed to the reconstruction of the surface to become as hydrophilic as a PEGylated surface when immersed in water [95].

Crosslinked terpolymer networks consisting of fluoropolymer, PDMS and PEG were also synthesized [96]. When evaluated for nonspecific protein resistance, the surface modified with the terpolymer was about 60% less susceptible to protein adhesion than that coated with PDMS.

## 4. Conclusions

This review provides a comprehensive view of chemical modification of the membrane surface to mitigate fouling for wastewater treatment. Specifically, we have reviewed key strategies in designing materials with antifouling properties to be coated or grafted on the membrane surface to mitigate fouling and retain high water permeance. Most of the materials are hydrophilic, such as PEG, polydopamine and zwitterions, which form tight hydration layers on the surface acting as a physical and energy barrier preventing foulants from attaching to the membrane surface. The grafted polymer chains on the membrane surface may also have repulsive elastic force against the adhesion of foulants. Hydrophobic materials such as perfluoropolymers have also been used as fouling-resistant layers due to their non-stick characteristic. Amphiphilic materials containing hydrophilic PEG and hydrophobic fluoropolymers or PDMS have also demonstrated the synergistic effect in achieving superior antifouling properties.

While the surface modification of membranes using materials with antifouling properties has been shown to retain high water permeance, there is a lack of the fundamental understanding of the structure/property relationship for the coating materials and membrane performance. We describe the state of the water in polymers aiming to shed some light on the mechanism of the antifouling properties. We have also identified active areas that could be fruitful to further improve the effectiveness of membrane fouling mitigation, such as the design and synthesis of new dopamine-like materials and new zwitterionic materials. These material platforms may have an enormous possibility in chemical structures, rendering great promise for membrane surface modification to retain long-term stability.
